# Characterization of Type VI secretion system in *Edwardsiella ictaluri*

**DOI:** 10.1371/journal.pone.0296132

**Published:** 2023-12-28

**Authors:** Safak Kalindamar, Hossam Abdelhamed, Adef O. Kordon, Hasan C. Tekedar, Lesya Pinchuk, Attila Karsi

**Affiliations:** 1 Department of Molecular Biology and Genetics, Faculty of Arts and Sciences, Ordu University, Ordu, Türkiye; 2 Department of Comparative Biomedical Sciences, College of Veterinary Medicine, Mississippi State University, Mississippi State, MS, United States of America; Federal University Dutse, NIGERIA

## Abstract

*Edwardsiella ictaluri* is a Gram-negative facultative intracellular fish pathogen causing enteric septicemia of catfish (ESC). While various secretion systems contribute to *E*. *ictaluri* virulence, the Type VI secretion system (T6SS) remains poorly understood. In this study, we constructed 13 *E*. *ictaluri* T6SS mutants using splicing by overlap extension PCR and characterized them, assessing their uptake and survival in channel catfish (*Ictalurus punctatus*) peritoneal macrophages, attachment and invasion in channel catfish ovary (CCO) cells, in vitro stress resistance, and virulence and efficacy in channel catfish. Among the mutants, *Ei*Δ*evpA*, *Ei*Δ*evpH*, *Ei*Δ*evpM*, *Ei*Δ*evpN*, and *Ei*Δ*evpO* exhibited reduced replication inside peritoneal macrophages. *Ei*Δ*evpM*, *Ei*Δ*evpN*, and *Ei*Δ*evpO* showed significantly decreased attachment to CCO cells, while *Ei*Δ*evpN* and *Ei*Δ*evpO* also displayed reduced invasion of CCO cells (*p* < 0.05). Overall, T6SS mutants demonstrated enhanced resistance to oxidative and nitrosative stress in the nutrient-rich medium compared to the minimal medium. However, *Ei*Δ*evpA*, *Ei*Δ*evpH*, *Ei*Δ*evpM*, *Ei*Δ*evpN*, and *Ei*Δ*evpO* were susceptible to oxidative stress in both nutrient-rich and minimal medium. In fish challenges, *Ei*Δ*evpD*, *Ei*Δ*evpE*, *Ei*Δ*evpG*, *Ei*Δ*evpJ*, and *Ei*Δ*evpK* exhibited attenuation and provided effective protection against *E*. *ictaluri* wild-type (*Ei*WT) infection in catfish fingerlings. However, their attenuation and protective efficacy were lower in catfish fry. These findings shed light on the role of the T6SS in *E*. *ictaluri* pathogenesis, highlighting its significance in intracellular survival, host cell attachment and invasion, stress resistance, and virulence. The attenuated T6SS mutants hold promise as potential candidates for protective immunization strategies in catfish fingerlings.

## Introduction

*Edwardsiella ictaluri* was initially isolated from channel catfish (*Ictalurus punctatus*) [1, 2], and is a significant fish pathogen causing acute septicemia or chronic encephalitis [3–5]. This bacterium has evolved remarkable adaptations to thrive within catfish phagocytic cells, such as macrophages and neutrophils [5–7]. *E*. *ictaluri* has been found to survive in various host immune cells, including catfish head kidney and peritoneal macrophages [8, 9] as well as neutrophils [10]. Despite these observations, the precise mechanisms employed by *E*. *ictaluri* for intracellular survival remain largely unexplored.

*Edwardsiella ictaluri* employs various defense mechanisms to adapt to intracellular stress. Among these mechanisms, the Type III Secretion System (T3SS) has been identified as crucial for replication within catfish macrophages [11]. The secretion of T3SS effector proteins plays a vital role in facilitating bacterial replication within the macrophage environment [12, 13]. Notably, mutations in T3SS effector genes have been found to impair the intracellular replication of *E*. *ictaluri* [14]. In addition to the T3SS, several genes have been identified as potentially significant for the survival of *E*. *ictaluri* inside neutrophils. These genes include enzymes involved in the tricarboxylic acid cycle (TCA), glycine cleavage system, sigmaE (σ^E^) regulator, the SoxS oxidative response system, and a plasmid-encoded T3SS effector [15].

The Type VI Secretion System (T6SS) is a sophisticated protein complex resembling a needle that facilitates the transport of effector proteins across the cell membrane in Gram-negative bacteria [16]. Structurally, the T6SS exhibits homology with the puncturing device found in bacteriophage T4 [17]. The core components, consisting of thirteen conserved genes, are essential for the functionality of the T6SS [18]. In the *E*. *ictaluri* strain 93–146 genome, the T6SS is encoded by an operon comprising 16 genes, namely *evpPABCDEFGHIJKLMNO* [19, 20]. The core genes involved in T6SS assembly are classified as membrane-associated proteins (*evpN*, *evpO*, *evpL*, and *evpM*) and bacteriophage T4 phage-related proteins (*evpK*, *evpA*, *evpB*, *evpC*, *evpE*, *evpI*, *evpH*, *evpF*, and *evpG*) [21].

Initially, several T6SS proteins, including Eip19 (*evpE*), Eip18 (*evpC*), Eip55 (*evpB*), and Eip20 (*evpA*) were identified in *E*. *ictaluri* during catfish infection [22]. The transcriptional regulation of the T6SS operon is governed by the Ara-C type regulatory protein EsrC, which is part of the EsrA-EsrB two-component system (TCs). EsrC acts as a sensor for environmental changes, such as variations in pH and inorganic phosphate (Pi) concentrations, thereby controlling the expression of the T6SS [11]. Additionally, the transcription of *evpP*, a component of the T6SS, is regulated by the Ferric uptake regulator (Fur) protein, which binds to the Fur box in the *evpP* promoter [23]. The T6SS in *E*. *ictaluri* serves a dual role, facilitating both survival within host cells and competition against inter-bacterial and intra-bacterial species. This versatility is achieved by delivering effector proteins into eukaryotic or prokaryotic cells [24].

The critical role of the T6SS in the *Edwardsiella* genus was initially demonstrated in the fish pathogen *Edwardsiella piscicida* [18, 25]. Secretion of EvpC, EvpI, and EvpP proteins has been observed, and mutations in T6SS genes, except evpD, resulted in attenuated virulence in the host [18]. Evidence suggests a potential interaction between the secreted protein evpC and a disordered region of evpP, indicating that evpP may serve as a secreted effector protein primarily targeting inflammasome activation in macrophages [26, 27]. In *E*. *ictaluri*, *evpP* has been shown to induce increased necrosis in anterior kidney macrophages [28]. The activation and repression of T6SS in *E*. *piscicida* are influenced by various environmental factors, including temperature, pH, Mg^2+^, P_i_, and iron availability [29, 30]. Our previous study revealed that *hcp1* (*evpC*) and *hcp2* are involved in virulence, adhesion to epithelial cells, and replication within catfish peritoneal macrophages, further emphasizing the importance of T6SS in the pathogenicity of *E*. *ictaluri* [31].

This study aimed to investigate the multifaceted role of the T6SS in *E*. *ictaluri*. Specifically, our objectives encompassed understanding the impact of T6SS on various important aspects, including survival and replication within macrophages, adhesion and invasion to catfish epithelial cells, adaptation and resilience to oxidative stress, as well as its contribution to virulence in catfish fingerlings and fry. By investigating the role of T6SS in the survival and replication of *E*. *ictaluri* within macrophages aids in unraveling the strategies employed by the bacterium to evade the host immune response and establish a persistent infection. This aspect highlights the interplay between the T6SS and the host’s immune defense mechanisms. In addition, examining the involvement of T6SS in adhesion and invasion to catfish epithelial cells, we sought to elucidate the mechanisms through which *E*. *ictaluri* interacts with and establishes infection in host tissues. Understanding this aspect of the T6SS function provides valuable insights into the initial stages of pathogenesis. Furthermore, we aimed to explore the contribution of T6SS to the adaptation and survival of *E*. *ictaluri* under oxidative stress conditions. Oxidative stress is a significant challenge faced by bacteria during infection, and understanding the role of T6SS in oxidative stress resistance provides insights into the survival strategies of *E*. *ictaluri* within the host environment. Finally, assessing the impact of T6SS on the virulence of *E*. *ictaluri* in catfish fingerlings and fry enables us to establish the link between T6SS functionality and disease severity. By unraveling the specific contributions of T6SS to the pathogenicity of *E*. *ictaluri*, we can identify potential targets for therapeutic interventions and develop strategies to mitigate the impact of disease in aquaculture.

## Materials and methods

### Ethics statement

This study was carried out in strict accordance with the recommendations in the Guide for the Care and Use of Laboratory Animals of the National Institutes of Health. The protocol was approved by the Institutional Animal Care and Use Committee of the Mississippi State University (Protocol Number: 15–043). Catfish peritoneal macrophages were collected under tricaine methanesulfonate anesthesia (100 mg/ml), and all catfish used in the study were euthanized using a high dose of tricaine methanesulfonate (400 mg/ml). All efforts were made to minimize suffering.

### Bacteria, plasmids, and growth conditions

Bacterial strains and the plasmid used in this work are listed in Table 1. The *E*. *ictaluri* strain 93–146 (*Ei*WT) was cultivated at 30°C either in brain-heart infusion (BHI) broth for 16 h or on BHI agar plates for 2 days. *Escherichia coli* CC118λ*pir* and BW19851 (Δ*uidA*3::*pir*) strains were cultured in Lysogeny broth (LB) or on Lysogeny agar (LA) at 37^o^ C for 16 h. Antibiotics were incorporated into the culture medium, with final concentrations as follows: ampicillin (100 μg/ml), colistin (12.5 μg/ml), and gentamicin (10 μg/ml in macrophage culture or 100 μg/ml to kill non-phagocyted *E*. *ictaluri*). Ampicillin and colistin were employed during the conjugation process. Ampicillin was used to select for *E*. *ictaluri* with the suicide plasmid, while colistin eliminated donor *E*. *coli* cells. Gentamicin was utilized in the bacterial killing assay to eliminate non-phagocytosed *E*. *ictaluri* selectively.

**Table 1:** Bacterial strains and plasmids.

Strain or plasmid	Description	Reference
***Edwardsiella ictaluri* wild-type**		
93–146	pEI1; pEI2; Col^r^	[58]
***Edwardsiella ictaluri* in-frame deletion mutants**		
*Ei*Δ*evpA*	93–146 derivative; pEI1; pEI2; Col^r^, Δ*evpA*	This study
*Ei*Δ*evpD*	93–146 derivative; pEI1; pEI2; Col^r^, Δ*evpD*	This study
*Ei*Δ*evpE*	93–146 derivative; pEI1; pEI2; Col^r^, Δ*evpE*	This study
*Ei*Δ*evpF*	93–146 derivative; pEI1; pEI2; Col^r^, Δ*evpF*	This study
*Ei*Δ*evpG*	93–146 derivative; pEI1; pEI2; Col^r^, Δ*evpG*	This study
*Ei*Δ*evpH*	93–146 derivative; pEI1; pEI2; Col^r^, Δ*evpH*	This study
*Ei*Δ*evpI*	93–146 derivative; pEI1; pEI2; Col^r^, Δ*evpI*	This study
*Ei*Δ*evpJ*	93–146 derivative; pEI1; pEI2; Col^r^, Δ*evpJ*	This study
*Ei*Δ*evpK*	93–146 derivative; pEI1; pEI2; Col^r^, Δ*evpK*	This study
*Ei*Δ*evpL*	93–146 derivative; pEI1; pEI2; Col^r^, Δ*evpL*	This study
*Ei*Δ*evpM*	93–146 derivative; pEI1; pEI2; Col^r^, Δ*evpM*	This study
*Ei*Δ*evpN*	93–146 derivative; pEI1; pEI2; Col^r^, Δ*evpN*	This study
*Ei*Δ*evpO*	93–146 derivative; pEI1; pEI2; Col^r^, Δ*evpO*	This study
***Escherichia coli* donors for conjugation**		
CC118λ*pir*	Δ*(ara-leu); araD;* Δ*lacX74; galE; galK; phoA20; thi-1; rpsE; rpoB; argE(Am); recAl; λpirR6K*	[59]
SM10λ*pir*	*thi; thr; leu; tonA; lacY; supE; recA;*::*RP4-2-Tc*::*Mu; Kmr; lpirR6K*	[60]
BW19851λ*pir*	*RP4-2 (Km*::*Tn7*, *Tc*::*Mu-1)*, *DuidA3*::*pir+*, *recA1*, *endA1*, *thi-1*, *hsdR17*, *creC510*	[61]
DH5α	dlacZ Delta M15 Delta(lacZYA-argF) U169 recA1 endA1 hsdR17(rK-mK+) supE44 thi-1 gyrA96 relA1 (2)	[62]
**Suicide plasmid**		
pMEG375	8142 bp, Amp^r^, Cm^r^, lacZ, R6K ori, mob incP, sacR sacB	[63]
**Suicide plasmids with in-frame deleted genes**		
pEiΔ*evpA*	9939 bp, pMEG-375, Δ*evpA*	This study
pEiΔ*evpD*	9939 bp, pMEG-375, Δ*evpD*	This study
pEiΔ*evpE*	9939 bp, pMEG-375, Δ*evpE*	This study
pEiΔ*evpF*	9939 bp, pMEG-375, Δ*evpF*	This study
pEiΔ*evpG*	9939 bp, pMEG-375, Δ*evpG*	This study
pEiΔ*evpH*	9939 bp, pMEG-375, Δ*evpH*	This study
pEiΔ*evpI*	9939 bp, pMEG-375, Δ*evpI*	This study
pEiΔ*evpJ*	9939 bp, pMEG-375, Δ*evpJ*	This study
pEiΔ*evpK*	9939 bp, pMEG-375, Δ*evpK*	This study
pEiΔ*evpL*	9939 bp, pMEG-375, Δ*evpL*	This study
pEiΔ*evpM*	9939 bp, pMEG-375, Δ*evpM*	This study
pEiΔ*evpN*	9939 bp, pMEG-375, Δ*evpN*	This study
pEiΔ*evpO*	9939 bp, pMEG-375, Δ*evpO*	This study
**Plasmid with bacterial *lux* and *gfp***		
pAK*gfplux1*	5681bp, PstI, EcoRI, HpaI, AseI, BstBI	[34]

### In-frame deletion of T6SS genes

The nucleotide sequences of the T6SS genes in *E*. *ictaluri* were acquired from the genomic data of *E*. *ictaluri* strain 93–146 [32]. To generate T6SS mutants in *E*. *ictaluri*, splicing by overlap extension PCR was employed. In brief, external and internal primer pairs were designed to amplify the upstream and downstream regions of each target gene (Table 2). The resulting two fragments were merged through the process of splicing by overlap extension (SOEing) [33]. The overlap extension PCR product was transferred to *E*. *ictaluri* by using plasmid pMEG375. Both the mutated insert (produced through overlap PCR) and pMEG375 underwent digestion with the same restriction enzymes, and the insert was subsequently ligated into pMEG375. Following electroporation and the selection of the correct plasmid in *E*. *coli* CC118, this plasmid was transferred to *E*. *coli* λ*pir* strains via electroporation. These *E*. *coli* λ*pir* strains were then utilized to transfer the plasmid into the *E*. *ictaluri* strain 93–146 through conjugation. A two-step selection process was implemented to obtain in-frame deletion mutants. In the first step, the conjugation mixture was inoculated in BHI broth containing ampicillin (selects for *E*. *ictaluri* with suicide plasmid) and colistin (eliminates donor *E*. *coli*). In the second step, positive colonies were streaked on BHI agar supplemented with colistin only. These colonies were re-streaked on the BHI agar containing 5% sucrose, 0.35% D-mannitol, and colistin. The colonies that exhibited sensitivity to ampicillin and featured the mutant band were identified as in-frame deletion colonies. The deletion of each gene was confirmed through PCR and sequencing.

**Table 2:** Primers used for in-frame deletion.

Primers	Sequence (5’ to 3’)^a^	RE^b^
*Ei*Δ*evpA*EF01	cccc**gcggccgc**ATCCCAGTAGGCATATATTGTTG	*Not*I
*Ei*Δ*evpA*IR01	tctgttcgctcattattgctgTCCGTAACATTTCTTACAACACC	
*Ei*Δ*evpA*IF01	CAGCAATAATGAGCGAACAGA	
*Ei*Δ*evpA*ER01	cccc**ggatcc**GGTACCCTTACAGTGGGTCAG	*Bam*HI
*Ei*Δ*evpD*EF01	cccc**tctaga**GCCCAAGGAATATGACAGTGA	*Xba*I
*Ei*Δ*evpD*IR01	gactgccagcgttttcagataCCGCTTGTCATCATCAGTGAG	
*Ei*Δ*evpD*IF01	TATCTGAAAACGCTGGCAGTC	
*Ei*Δ*evpD*ER01	cccc**gagctc**TCCCAGGGTATTCAGATGATG	*Sac*I
*Ei*Δ*evpE*EF01	cccc**tctaga**GTTCGATTCAACATCCTTTGG	*Xba*I
*Ei*Δ*evpE*IR01	tcacctgtctattcggtacagGGCTCAACTCACGGGATTGTC	
*Ei*Δ*evpE*IF01	CTGTACCGAATAGACAGGTGA	
*Ei*Δ*evpE*ER01	cccc**gagctc**TCGGGAATAATTTGGTACTCG	*Sac*I
*Ei*Δ*evpF*EF01	cccc**tctaga**GACCCAGCAGATTATCAATGC	*Xba*I
*Ei*Δ*evpF*IR01	ctgctgggtgcgtacatgCGCCAGTTCCCGGTTATAGTAG	
*Ei*Δ*evpF*IF01	CATGTACGCACCCAGCAG	
*Ei*Δ*evpF*ER01	cccc**gagctc**AAGTCACATCGCGTGGTAGG	*Sac*I
*Ei*Δ*evpG*EF01	cccc**tctaga**CCTGACCAAACTGACCGATA	*Xba*I
*Ei*Δ*evpG*IR01	ctgtgcacccgatttatcgCATGACAGCACCGCCTTA	
*Ei*Δ*evpG*IF01	CGATAAATCGGGTGCACAG	
*Ei*Δ*evpG*ER01	cccc**gagctc**GTACCGGCTTCAGCAGATTG	*Sac*I
*Ei*Δ*evpH*EF01	cccc**tctaga**TTTTCAGGCCGTCAGGATAC	*Xba*I
*Ei*Δ*evpH*IR01	ccatccttcactcccctctagGCAGGTCTGGTTCATTTTCC	
*Ei*Δ*evpH*IF01	CTAGAGGGGAGTGAAGGATGG	
*Ei*Δ*evpH*ER01	cccc**gagctc**ATTTAGGCAGGCAGACGAAG	*Sac*I
*Ei*Δ*evpI*EF01	cccc**tctaga**GTGCAGACCTGTGTGGATGA	*Xba*I
*Ei*Δ*evpI*IR01	cctttgatggaggtcatcacGGTGGATAAGGACAATAGCCG	
*Ei*Δ*evpI*IF01	GTGATGACCTCCATCAAAGG	
*Ei*Δ*evpI*ER01	cccc**gagctc**TGATGGCTTGTTGAAGATCG	*Sac*I
*Ei*Δ*evpJ*EF01	cccc**tctaga**TACCAGAACCACTTCGTCTGC	*Xba*I
*Ei*Δ*evpJ*IR01	ccttaaccgccaatcaacacCCAATACCATACCGCAGAGC	
*Ei*Δ*evpJ*IF01	GTGTTGATTGGCGGTTAAGG	
*Ei*Δ*evpJ*ER01	cccc**gagctc**TAACCCAATACACCCTTGAGC	*Sac*I
*Ei*Δ*evpK*EF01	cccc**tctaga**GTCTATAACGCCAACCAGACG	*Xba*I
*Ei*Δ*evpK*IR01	ttcatccagtctcatattgccACTGATGGGAGCCAGTAAACG	
*Ei*Δ*evpK*IF01	GGCAATATGAGACTGGATGAA	
*Ei*Δ*evpK*ER01	cccc**gagctc**TTAGCCCCAGGTAGACATTGA	*Sac*I
*Ei*Δ*evpL*EF01	cccc**tctaga**GTGTTGATTGGCGGTTAAGG	*Xba*I
*Ei*Δ*evpL*IR01	tccactgatatcgctcagccgGGTCAGACTGAGCAGCAGG	
*Ei*Δ*evpL*IF01	CGGCTGAGCGATATCAGTGGA	
*Ei*Δ*evpL*ER01	cccc**gagctc**CGAATAATCAGATCGCGTACC	*Sac*I
*Ei*Δ*evpM*EF01	cccc**tctaga**GTGGAGGGAAAAGGGATGAC	*Xba*I
*Ei*Δ*evpM*IR01	tcgaaccagctgaatgcaatCTGTAAAAACATGCCCTGATGC	
*Ei*Δ*evpM*IF01	ATTGCATTCAGCTGGTTCGA	
*Ei*Δ*evpM*ER01	cccc**gagctc**TACCGTCATGCATCTCATGG	*Sac*I
*Ei*Δ*evpN*EF01	cccc**tctaga**CCGACCGTTCAATGTCTACC	*Xba*I
*Ei*Δ*evpN*IR01	cgacatccgcatactcatataaGAGCATGGGCTGAATAAATGC	
*Ei*Δ*evpN*IF01	TTATATGAGTATGCGGATGTCG	
*Ei*Δ*evpN*ER01	cccc**gagctc**CGGTAGGGAACATCAATAGGG	*Sac*I
*Ei*Δ*evpO*EF01	cccc**tctaga**ATTTCCTGATCACCCTTGAGC	*Xba*I
*Ei*Δ*evpO*IR01	gcttgtcggttaactgtttggcAATCAGTGCAATCGCTATCC	
*Ei*Δ*evpO*IF01	GCCAAACAGTTAACCGACAAGC	
*Ei*Δ*evpO*ER01	cccc**gagctc**CAGCCGGTACCACAGGATCT	*Sac*I

^a^ Bold letters show restriction enzyme recognition sequences added to primers. Underlined letters indicate reverse complemented primer sequences.

^b^ Restriction enzyme.

### Construction of bioluminescent strains

The creation of bioluminescent T6SS mutants followed a procedure previously documented [34]. Briefly, *E*. *coli* SM10λ*pir* harboring pAK*gfplux*1 and the mutant strains were cultured overnight and combined at a 1:2 ratio (donor: recipient). The resulting pellet was applied to a 0.45 μM filter paper on BHI agar and incubated at 30°C for 24 h. The filter paper, containing a mixture of bacteria, was rinsed with BHI broth containing ampicillin and colistin, and serial dilutions were spread onto selective BHI agar containing ampicillin and colistin. Following incubation at 30°C for 24–48 h, ampicillin-resistant mutant colonies carrying pAK*gfplux*1 emerged on the selective plates.

### Bacterial killing assay

The bacterial killing assay was conducted following established procedures as outlined in previous references [31, 35–37]. In brief, 1 ml squalene (Sigma-Aldrich) was administered to a year-old sedated (100 mg/ml tricaine methanesulfonate) channel catfish (ABW = 250–300 g) to activate catfish peritoneal macrophages. After 4 days of injection, sterile and cold phosphate-buffered saline (PBS) was injected into the intraperitoneal area, and the cell suspension was collected into tubes on ice. Additional PBS was injected into the peritoneal cavity until the fluid became clear, and subsequently, the catfish were humanely euthanized (400 mg/ml tricaine methanesulfonate). Peritoneal macrophages (5 x 10^5^ cells) were harvested and mixed with bioluminescent *E*. *ictaluri* strains at a 1:1 ratio in a 96-well plate (Evergreen Scientific). Each well contained a final volume of 200 μl for the cell-bacteria mixture, and the plate included four replicate wells for each treatment and a negative control (containing only cells). The plate was centrifugated at 1500 rpm for 5 minutes at 24°C to compact the cells and bacteria at the bottom. Subsequently, the plate was incubated for 1 h at 30°C to facilitate the invasion of catfish peritoneal macrophages by bioluminescent mutants and *Ei*WT. After this initial incubation, the cell-bacteria mixture underwent centrifugation at 2000 rpm for 7 min, and the culture medium was aspirated. Fresh medium containing 100 μg/ml gentamicin was added, and the cells were further incubated for 1 additional h at 30°C to eliminate non-phagocyted *E*. *ictaluri*. Following this, each well was subjected to triple washes with PBS, and the peritoneal macrophages were resuspended in Channel Catfish Macrophage Medium supplemented with 10 μg/ml gentamicin. Finally, cells were transferred to black 96-well plates (Fisher Scientific), and the plate was placed in Cytation 5 instrument (BioTek), where the cells were incubated for 48 h under 5% CO_2_ at 30^o^ C. Bioluminescence data were recorded at hourly intervals, and subsequent analysis was performed to determine the number of surviving bioluminescent bacteria within catfish peritoneal macrophages.

### Attachment and invasion assays

Attachment and invasion assays were conducted using previously established procedures with some adjustments [38]. Briefly, channel catfish ovary (CCO) cells were suspended in Dulbecco’s Modified Eagle Medium (DMEM) (Sigma), supplemented with 10% fetal bovine serum and 4 mM L-glutamine, resulting in a final concentration of 1 x 10^7^ cells per ml. Bioluminescent mutants and *Ei*WT were combined with CCO cells at a 1:1 ratio and seeded in a 24-well plate. Each well held a final volume of 1 ml cell-bacteria mixture, and the plate included four replicate wells for each treatment, alongside a negative control with no bacteria. The plate was incubated at 28°C for 1 h to facilitate the attachment of mutants and *Ei*WT to CCO cells. Subsequently, the cell suspensions were exposed to DMEM containing 100 μg/ml gentamicin for 1 h to eliminate the externally located bacteria. The plate underwent three washes with PBS, and the bioluminescent emissions originating from the *E*. *ictaluri* strains were captured using the IVIS Lumina XRMS in Vivo Imaging System Series III and quantified utilizing Living Image Software version 4.7.4 (PerkinElmer).

### Stress assays

The resilience of the mutants to oxidative stress induced by hydrogen peroxide (H2O2) (Sigma) and nitrosative stress caused by sodium nitroprusside (SNP) (Sigma) was evaluated in both a rich medium (BHI) and a low phosphate minimal medium at pH 5.5 (MM19-P) [39]. Bacterial cultures were cultivated overnight, and OD_600_ was standardized to 0.5 for each culture. Subsequently, five μl of bacteria from each strain were introduced into 195 μl of BHI and MM19-P broth, each containing either 0.75 mM H_2_O_2_ (prepared from 30% stock solution) or 5 mM SNP. A 96-well black plate was employed for each stress condition, with three replicates allocated for each mutant, along with *Ei*WT as a positive control. After 4, 8, 12, and 24 h of incubation at 30°C, the average photon counts were determined using the IVIS Lumina XRMS in Vivo Imaging System Series III (PerkinElmer).

### Virulence and efficacy of T6SS mutants in catfish fingerlings and fry

Vaccination and efficacy assessments were conducted following previously established procedures [20]. In the vaccination stage, catfish are infected with mutants to determine their attenuation levels and vaccinate them. In the challenge stage, vaccinated catfish (catfish that survived after mutant infection) are infected with *E*. *ictaluri* wild-type (*Ei*WT) 21 days post-vaccination to assess efficacy. Briefly, specific-pathogen-free (SPF) channel catfish fingerlings and fry were obtained from the MSU-CVM Hatchery. Twenty-five catfish fingerlings (10.46 ± 0.86 cm, ABW = 14.03 ± 3.57 g) were stocked into each tank and acclimated at 26–28°C for one week. During acclimation and experiments, 12 h on and 12 h off light cycle was applied, catfish were fed twice a day, and chlorine, dissolved oxygen, and temperature were monitored daily. Tanks were randomly assigned to 13 T6SS mutants (vaccination), *Ei*WT (positive control), and BHI (sham) groups. Each treatment had three replicates. Immersion vaccination was applied by lowering the water level in each tank to 10-L and adding 100 ml of bacterial culture (final dose of 2.4 x 10^7^ CFU/ml water). After 1 h, water flow (1 liter/min) was restored to each tank. Infected fish were monitored twice a day, and dead fish were collected. In addition, fish exhibiting loss of equilibrium accompanied by disease-specific clinical signs, such as petechial hemorrhages, exophthalmia, swollen abdomen, and skin lesions, were removed immediately and euthanized (400 mg/ml tricaine methanesulfonate) to minimize suffering. Mortalities were recorded daily for 21 days, and the percent mortalities were calculated for each group. To assess the protective capabilities of mutants, all fish that survived the vaccination were challenged with *Ei*WT (2.8 x 10^7^ CFU/ml) 21 days post-vaccination, as described above. The experiment was terminated by euthanizing all fish using 400 mg/ml tricaine methanesulfonate when no fish mortalities were observed for three consecutive days. Virulence and efficacy of mutants exhibiting good attenuation and protection in fingerlings were tested in 14-day-old catfish fry (0.14 cm, ABW = 0.03 g) as described above.

### Statistical analysis

The significance of variations in means among treatment groups was determined using one-way ANOVA and two-way ANOVA procedures, followed by Tukey’s test for post hoc analysis. These analyses were conducted within the SAS for Windows 9.4 software (SAS Institute, Inc., Cary, NC). A significance threshold of *p* < 0.05 was adopted for all tests.

## Results

### Components of *E*. *ictaluri* T6SS

**Fig 1:**
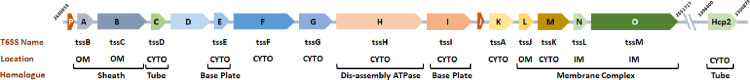
Gene organization of T6SS operon in *E*. *ictaluri* 93–146 genome. T6SS genes were represented with the common T6SS name (tss), proteins location (OM: Outer membrane, CYTO: Cytoplasmic, IM: Inner membrane), and their homolog of bacteriophage T4 (Baseplate, disassembly ATPase, sheath, and membrane complex). Gene sizes and distances were represented relatively.

*E*. *ictaluri* harbors a compact T6SS operon spanning 20,784 nucleotides, encompassing the *evpP* to *evpO* genes (Fig 1). Notably, in addition to this operon, an *hcp2* gene, which shares homology with *evpP*, was identified outside of the core T6SS operon.

### Phagocytic uptake and survival of the mutants in catfish peritoneal macrophages

**Fig 2:**
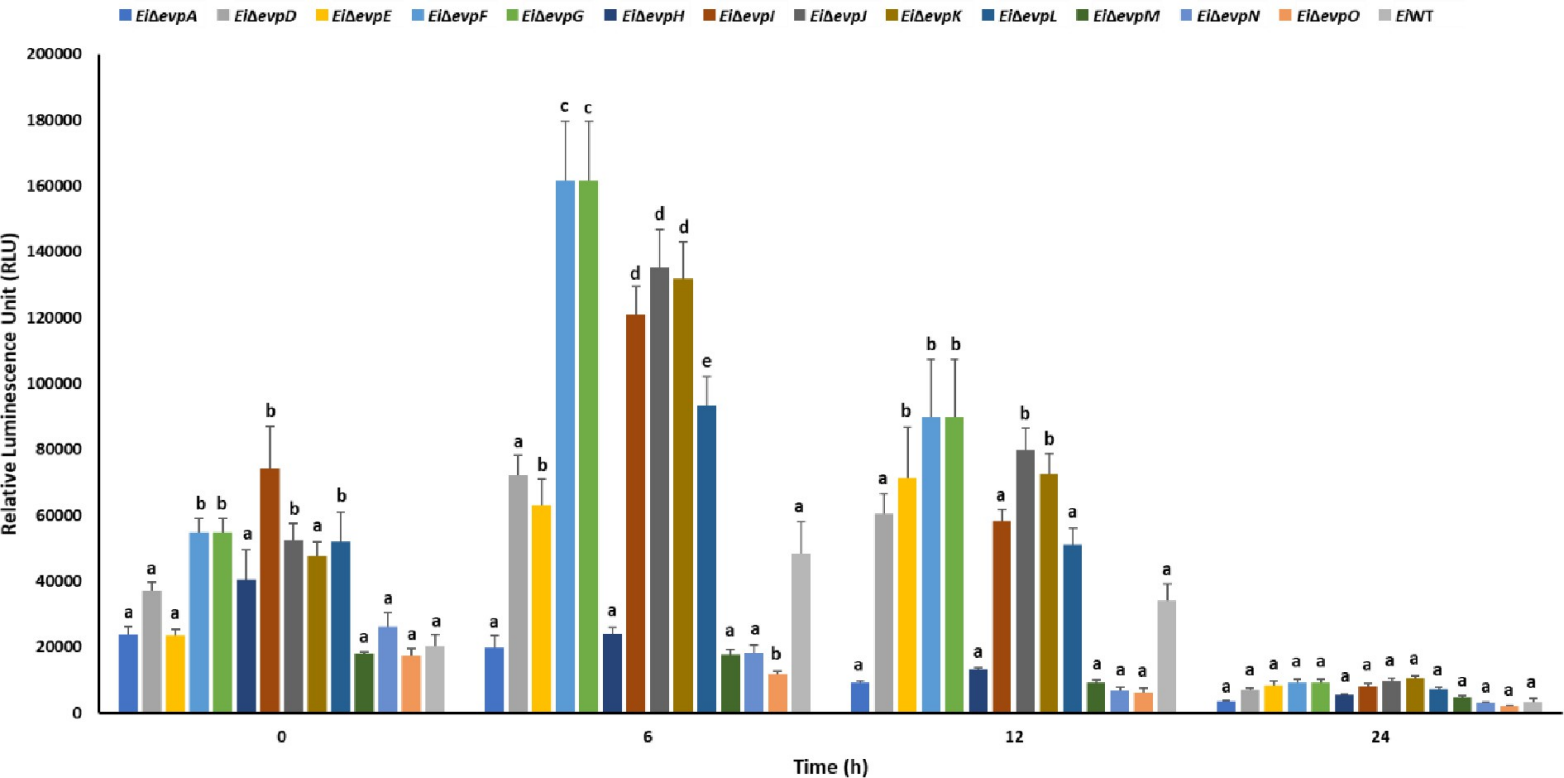
Bacterial killing assay of T6SS mutants and *Ei*WT. The mean photon emission was obtained from each mutant and positive (*Ei*WT) and negative (cell only) controls. The data represent the mean ±SD from one experiment. The experiment included four replicate wells for each mutant and control. Negative control was used to subtract background noise bioluminescence from each sample. The lowercase letters show significant differences between treatments (*p* < 0.05).

Uptake and intracellular survival of the T6SS mutants and WT within catfish peritoneal macrophages were summarized in Fig 2. In phagocytic uptake, *Ei*Δ*evpF*, *Ei*Δ*evpG*, *Ei*Δ*evpI*, *Ei*Δ*evpJ*, and *Ei*Δ*evpL* showed significantly higher internalization rates than other T6SS mutants and *Ei*WT at 0 h. At 6 hours, the numbers of intracellular *Ei*Δ*evpE*, *Ei*Δ*evpF*, *Ei*Δ*evpG*, *Ei*Δ*evpI*, *Ei*Δ*evpJ*, *Ei*Δ*evpK*, and *Ei*Δ*evpL* were significantly higher than *Ei*WT. *Ei*Δ*evpF* and *Ei*Δ*evpG* replicated in significantly higher rates than other mutants, and interestingly, *Ei*Δ*evpO* remained at a lower replication rate than *Ei*WT. *Ei*Δ*evpE*, *Ei*Δ*evpF*, *Ei*Δ*evpG*, *Ei*Δ*evpJ*, and *Ei*Δ*evpK* survived inside the catfish peritoneal macrophages up to 12 h. *Ei*Δ*evpL* had a higher phagocytic uptake rate at 0 h, but the number of intracellular *Ei*Δ*evpL* decreased after 6 h. Although *Ei*Δ*evpE* and *Ei*Δ*evpK* had similar phagocytic uptake as *Ei*WT, the number of intracellular *Ei*Δ*evpE* and *Ei*Δ*evpK* increased up to 12 h. At 24 h, the number of intracellular T6SS mutants and *Ei*WT were significantly decreased, and there were no significant differences among T6SS mutants and *Ei*WT. Together, these data indicated that *Ei*WT could replicate in catfish peritoneal macrophages for a limited time (6 h), and after this, macrophages kill *Ei*WT gradually. Deleting T6SS genes caused an increased uptake of some mutants by macrophages at 6 h, but the killing of mutants progressed at a pattern similar to *Ei*WT.

### Attachment and invasion of the mutants in CCO cells

**Fig 3:**
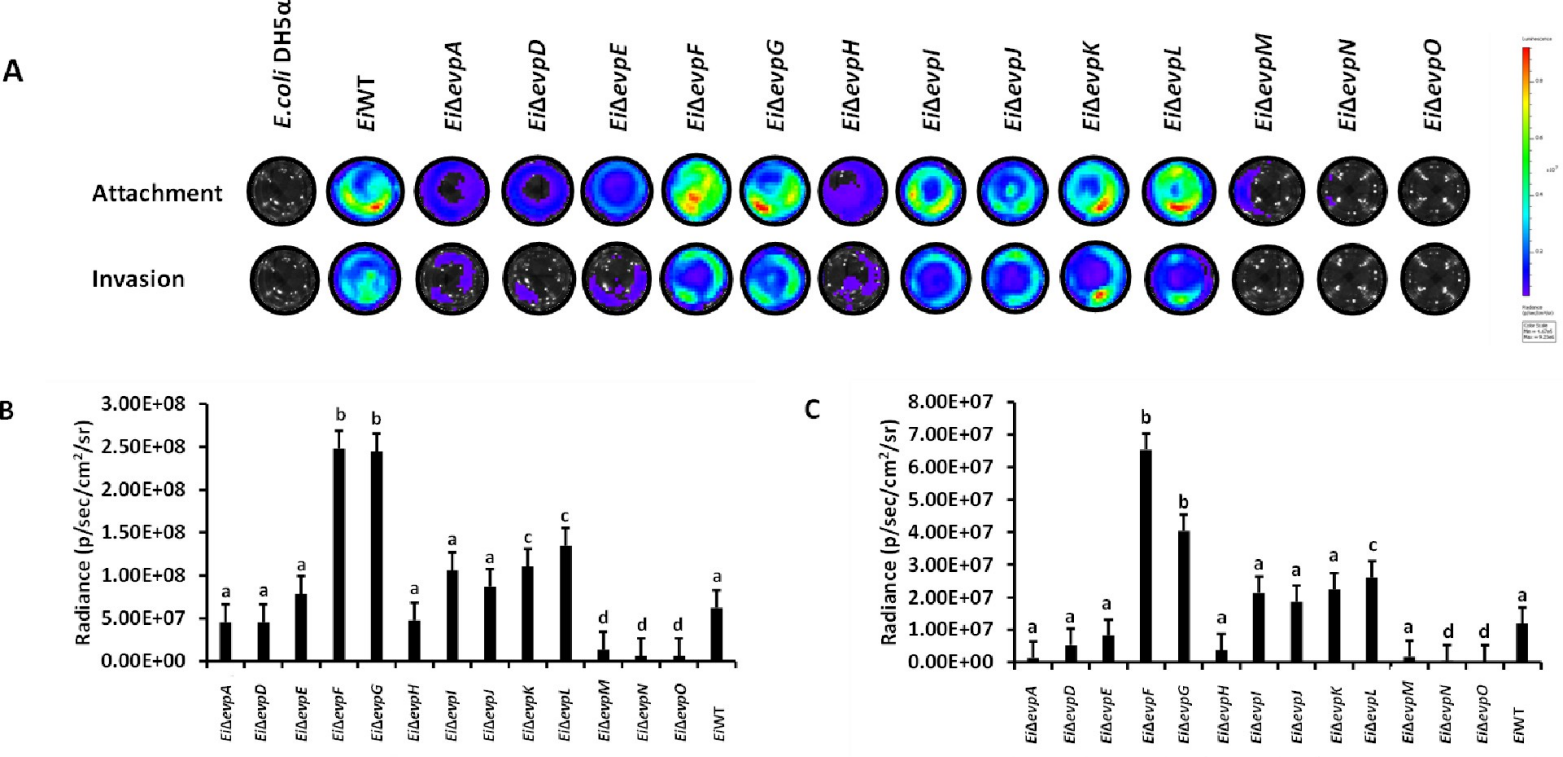
Attachment and invasion of T6SS mutants and *Ei*WT to CCO. The mean photon emission was obtained from each mutant and positive (*Ei*WT) and negative (*E*. *coli* DH5α and cell only) controls. The data represent the mean ±SD from one experiment. The experiment included three replicate wells for each mutant and control. Negative control was used to subtract background noise bioluminescence from each sample. **(A)** A representative image of bioluminescent imaging of CCO cells treated with bioluminescent T6SS mutants. **(B)** The mean photon emission shows the attachment ability of T6SS mutants and *Ei*WT. The lowercase letters show significant differences between treatments (*p* < 0.05). **(C)** The mean photon exposure was obtained from the same 24-well plate, including gentamycin, for an hour after attachment.

Attachment and invasion of T6SS mutants and *Ei*WT were assessed in CCO cells by bioluminescent imaging (Fig 3A). The blue and red colors on the scale indicate low to high bacterial loads, respectively. *Ei*Δ*evpF*, *Ei*Δ*evpG*, *Ei*Δ*evpK*, and *Ei*Δ*evpL* had significantly increased attachment rates compared to *Ei*WT (Fig 3B). On the other hand, attachments of *Ei*Δ*evpM*, *Ei*Δ*evpN*, and *Ei*Δ*evpO* were significantly lower than that of *Ei*WT. Invasion patterns were quite similar to attachment patterns: *Ei*Δ*evpF*, *Ei*Δ*evpG*, and *Ei*Δ*evpL* had significantly higher invasion rates compared to other mutants and *Ei*WT, while invasion capabilities of *Ei*Δ*evpN* and *Ei*Δ*evpO* were the lowest of all (Fig 3C).

### Survival and stress resistance of the mutants

**Fig 4:**
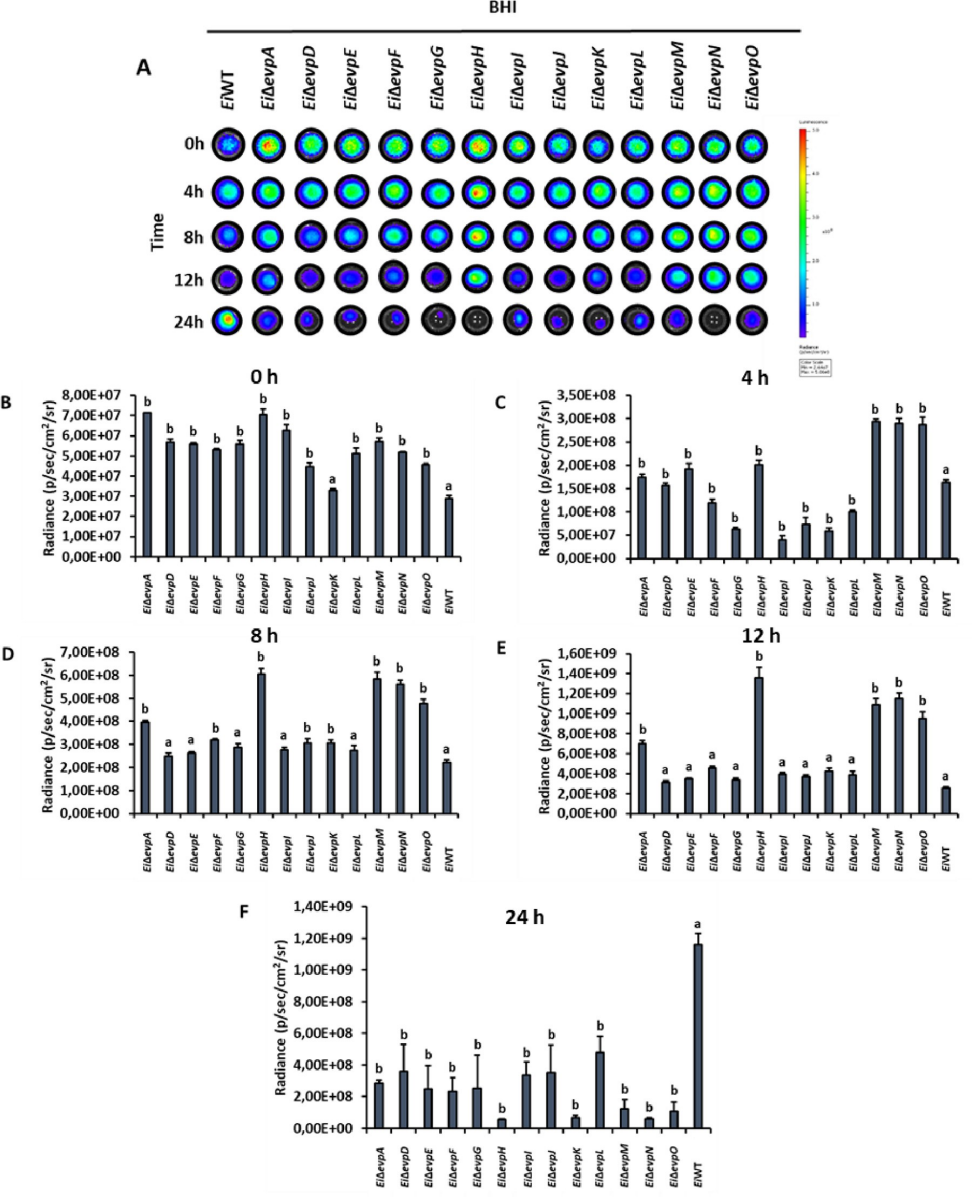
Stress assays of T6SS mutants and *Ei*WT exposed to SNP in BHI. The mean photon emission was obtained from each mutant and positive (*Ei*WT) and negative (no bacteria) controls. The data represent the mean ±SD from one experiment. The experiment included three replicate wells for each mutant and control. Negative control was used to subtract background noise bioluminescence from each sample. The lowercase letters show significant differences between treatments (*p* < 0.05). **(A)** Bioluminescent image of T6SS mutants and *Ei*WT exposed to SNP in BHI for 24 h. **(B ‐ F)** The bar graphs indicated the relative luminescence unit (RLU) obtained from the T6SS mutants and *Ei*WT treated with SNP in BHI for 0, 4, 8, 12, and 24 h.

**Fig 5:**
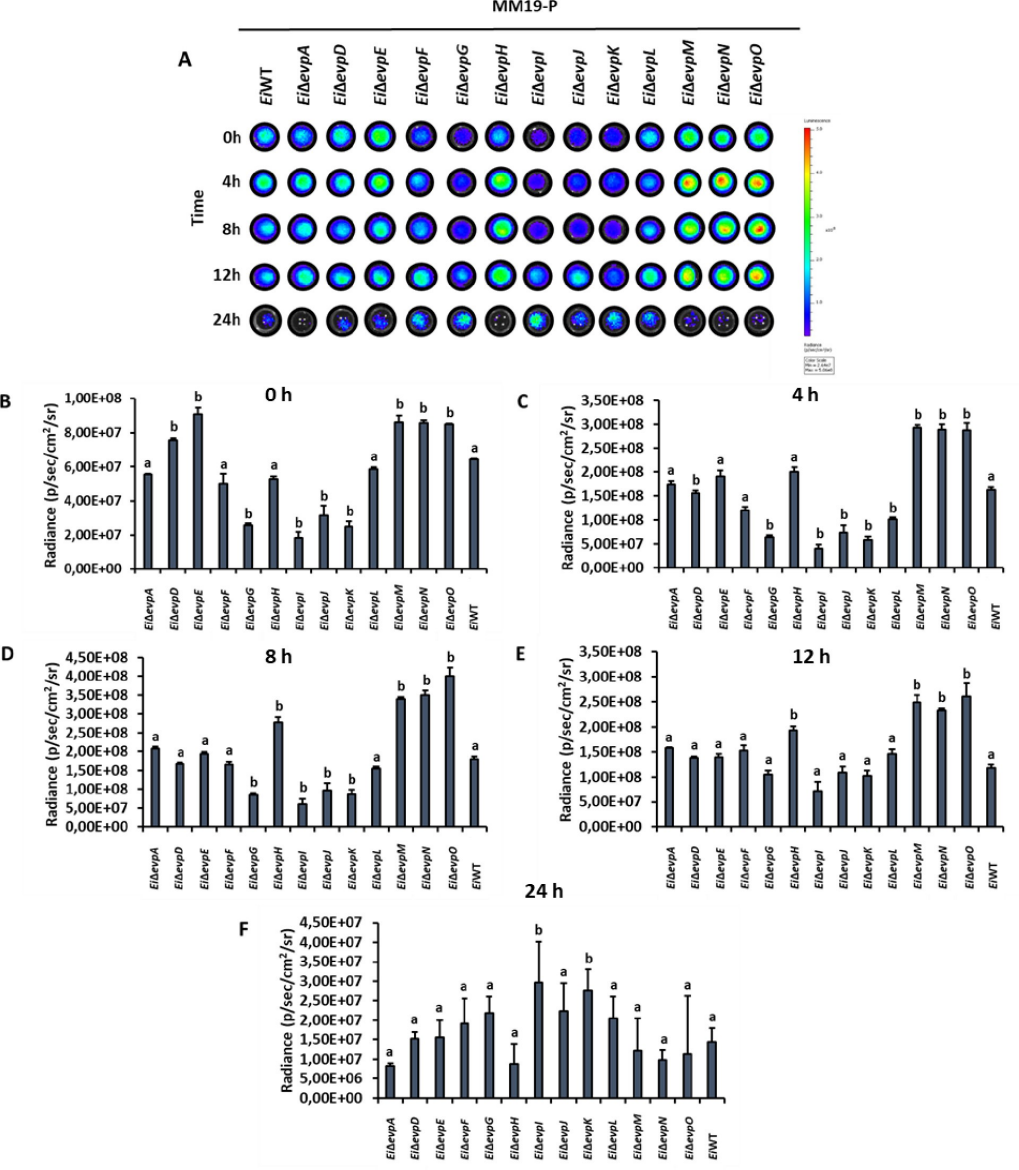
Stress assays of T6SS mutants and *Ei*WT exposed to SNP in MM19-P. The mean photon emission was obtained from each mutant and positive (EiWT) and negative (no bacteria) controls. The data represent the mean ±SD from one experiment. The experiment included three replicate wells for each mutant and control. Negative control was used to subtract background noise bioluminescence from each sample. The lowercase letters show significant differences between treatments (*p* < 0.05). **(A)** Bioluminescent image of T6SS mutants and *Ei*WT exposed to SNP in MM19-P for 24 h. **(B ‐ F)** The bar graphs indicated the relative luminescence unit (RLU) obtained from the T6SS mutants and *Ei*WT treated with SNP in MM19-P for 0, 4, 8, 12, and 24 h.

Innate immune cells can respond to pathogenic bacteria invasion by activating nitrosative and oxidative stress mechanisms. SNP and H_2_O_2_ stresses were applied to imitate the phagosomal stress conditions. The minimal medium (MM19-P) mimicked the nutrient-poor phagosomal conditions, in which T6SS mutants showed lower growth than BHI (Figs 4A and 5A). At 0–12 h, some T6SS mutants showed variable resistance to SNP in BHI, but growth of *Ei*WT was higher than all mutants at 24 h (Fig 4B–4F). Similarly, T6SS mutants showed variable resistance to SNP in MM19-P at 0–12 h, but growth of *Ei*Δ*evpI* and *Ei*Δ*evpK* was higher than all mutants and *Ei*WT (Fig 5B–5F).

**Fig 6:**
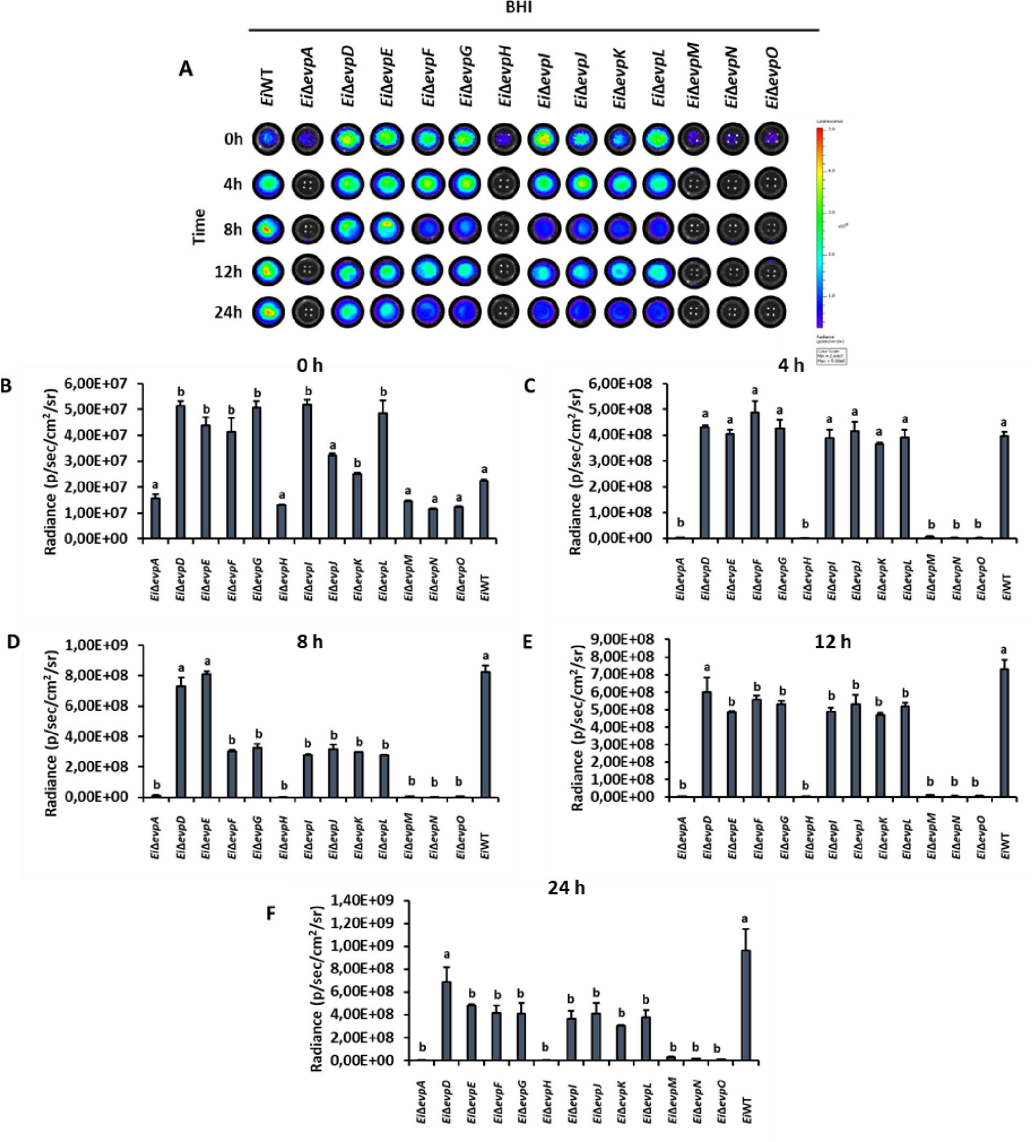
Stress assays of T6SS mutants and *Ei*WT exposed to H_2_O_2_ in BHI. The mean photon emission was obtained from each mutant and positive (*Ei*WT) and negative (no bacteria) controls. The data represent the mean ±SD from one experiment. The experiment included three replicate wells for each mutant and control. Negative control was used to subtract background noise bioluminescence from each sample. The lowercase letters show significant differences between treatments (*p* < 0.05). **(A)** Bioluminescent image of T6SS mutants and *Ei*WT exposed to H_2_O_2_ in BHI for 24 h. **(B ‐ F)** The bar graphs indicated the relative luminescence unit (RLU) obtained from the T6SS mutants and *Ei*WT treated with H_2_O_2_ in BHI for 0, 4, 8, 12, and 24 h.

**Fig 7:**
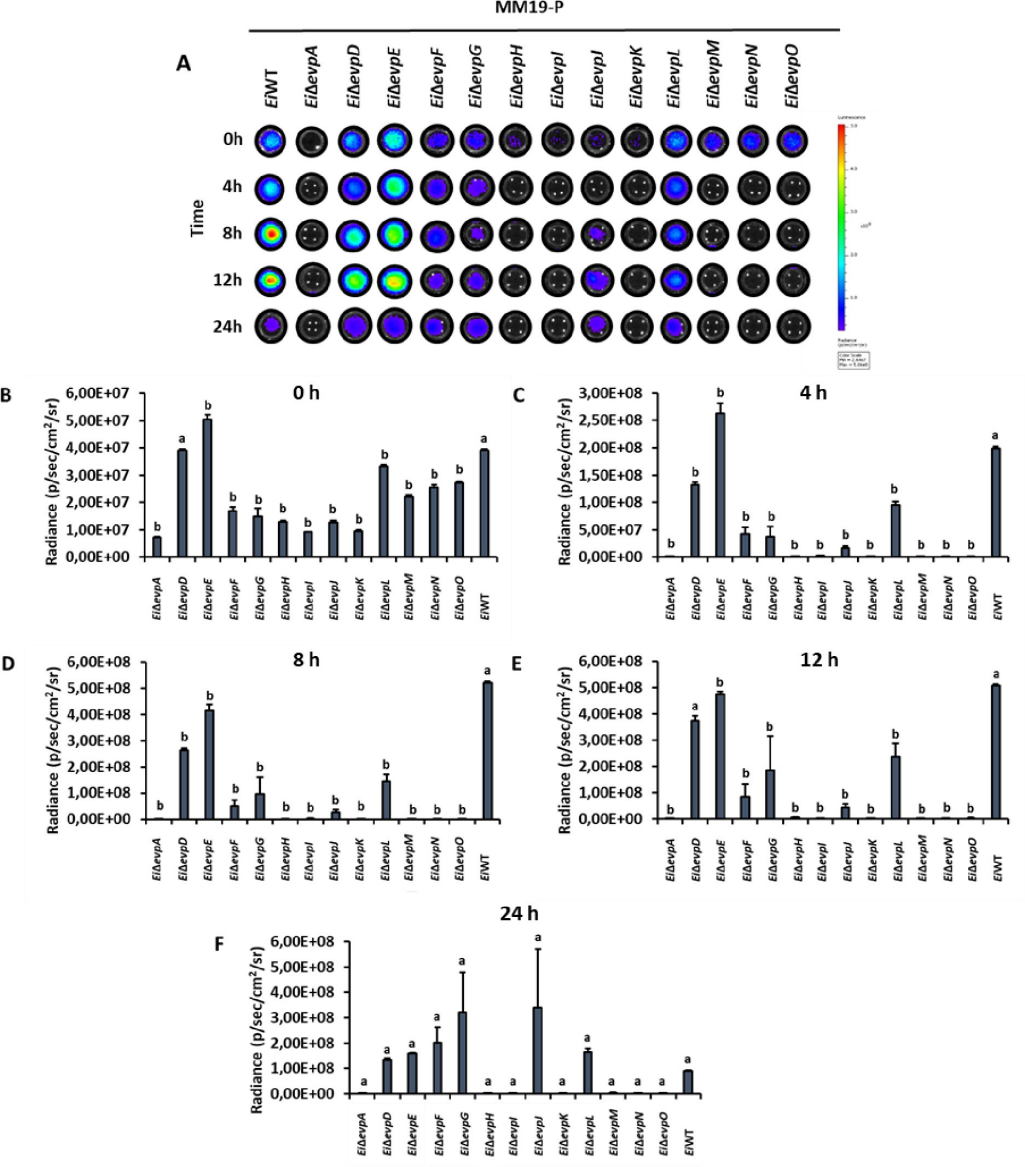
Stress assays of T6SS mutants and *Ei*WT exposed to H_2_O_2_ in MM19-P. The mean photon emission was obtained from each mutant and positive (*Ei*WT) and negative (no bacteria) controls. The data represent the mean ±SD from one experiment. The experiment included three replicate wells for each mutant and control. Negative control was used to subtract background noise bioluminescence from each sample. The lowercase letters show significant differences between treatments (*p* < 0.05). **(A)** Bioluminescent image of T6SS mutants and *Ei*WT exposed to H_2_O_2_ in MM19-P for 24 h. **(B ‐ F)** The bar graphs indicated the relative luminescence unit (RLU) obtained from the T6SS mutants and *Ei*WT treated with SNP in MM19-P for 0, 4, 8, 12, and 24 h.

The treatment of T6SS mutants with H_2_O_2_ caused reduced bacterial growth in both BHI and MM19-P (Figs 6A and 7A). Growth of *Ei*Δ*evpA*, *Ei*Δ*evpH*, *Ei*Δ*evpM*, *Ei*Δ*evpN*, and *Ei*Δ*evpO* was significantly affected by H_2_O_2_ stress in BHI, and at 24 h, growth of all mutants except *Ei*Δ*evpD* was lower than *Ei*WT (Fig 6B–6F). In MM19-P, H_2_O_2_ stress caused significant growth loss for *Ei*Δ*evpA*, *Ei*Δ*evpH*, *Ei*Δ*evpI*, *Ei*Δ*evpK*, *Ei*Δ*evpM*, *Ei*Δ*evpN*, and *Ei*Δ*evpO* by 24 h (Fig 7B–7F). These results show that T6SS mutants are more sensitive to SNP and H_2_O_2_ in MM19 due to the low pH (5.5) in MM19-P. The resistance of T6SS mutants to SNP is more than H_2_O_2_. Hydrogen peroxide can restrict the bacterial growth of T6SS mutants in both MM19-P and BHI. T6SS mutants are sensitive to stress factors that imitate phagosomal killing conditions inside catfish macrophages.

### Assessment of virulence and efficacy of the mutants in catfish

**Fig 8:**
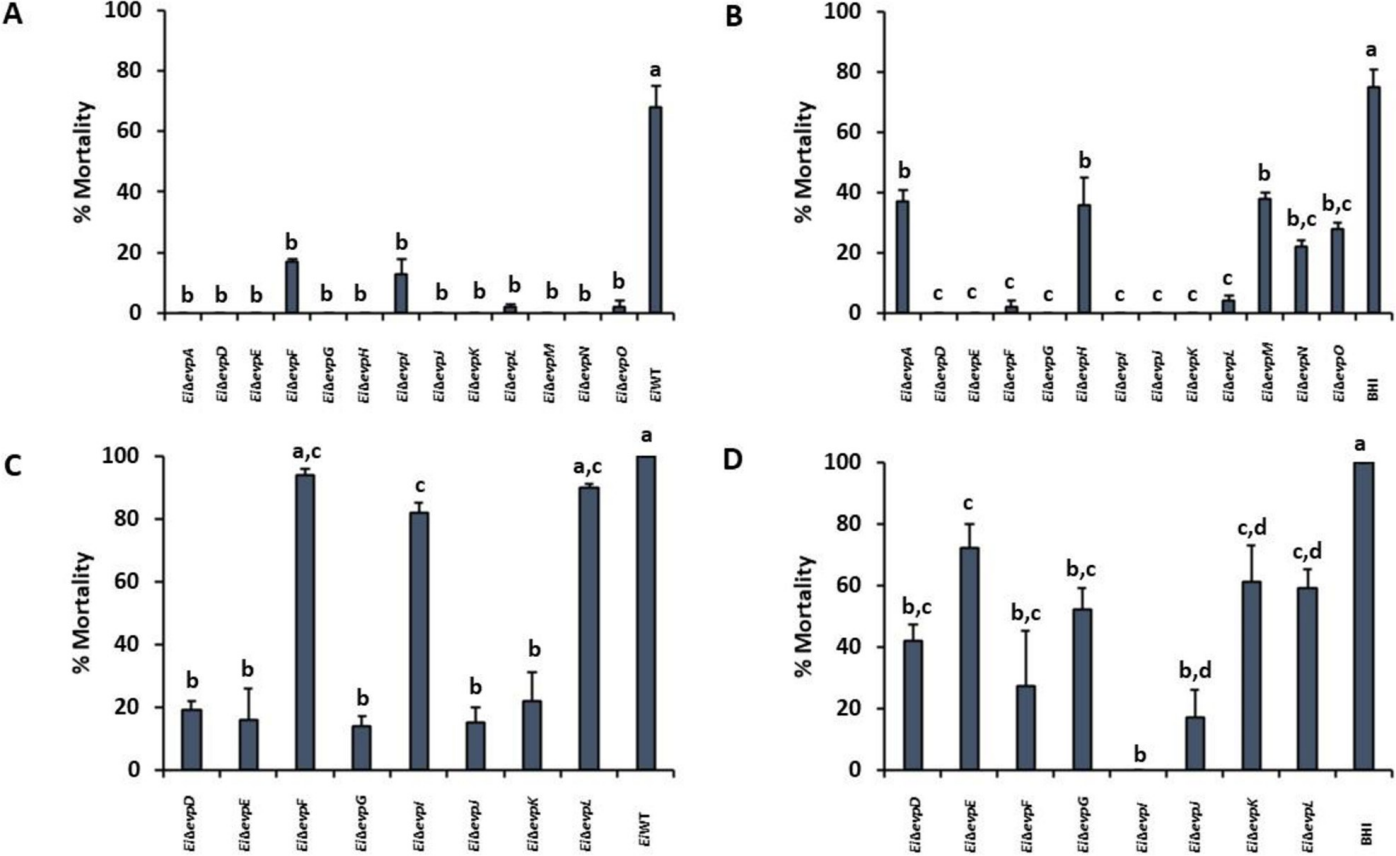
Attenuation, vaccination, and efficacy testing of T6SS mutants. In the vaccination stage, fish are infected with mutants to determine their attenuation levels and, at the same time, vaccinate fish. In the challenge stage, vaccinated fish (fish survived after mutant infection) are infected with *Ei*WT 21 days post-vaccination to assess efficacy. **(A)** virulence and **(B)** efficacy of T6SS mutants and *Ei*WT in catfish fingerlings. **(C)** virulence and **(D)** efficacy of T6SS mutants and *Ei*WT in catfish fry. The data represent the mean percent mortalities ±SD from one experiment. The experiment included three replicate fish tanks, each containing 25 fish. The letters above bars show statistical significance between treatments (*p* < .05).

All mutants were completely attenuated or significantly less virulent than *Ei*WT (68% mortality) in catfish fingerlings (Fig 8A) (*p* < 0.05). *Ei*Δ*evpF*, *Ei*Δ*evpI*, *Ei*Δ*evpL*, and *Ei*Δ*evpO* were attenuated but caused some mortality (16.95%, 13.18%, 1.75%, and 2.38% mortality, respectively). Infection of fish with *Ei*WT 21-days post-infection showed that all mutants provided better protection than sham vaccination (75% mortality), especially *Ei*Δ*evpD*, *Ei*Δ*evpE*, *Ei*Δ*evpG*, *Ei*Δ*evpJ*, and *Ei*Δ*evpK* were quite efficacious (Fig 8B). Although completely attenuated, *Ei*Δ*evpA*, *Ei*Δ*evpH*, *Ei*Δ*evpM*, and *Ei*Δ*evpN* caused less protection (37.42%, 35.80%, 37.78%, and 21.80% mortality, respectively) (Fig 8B).

Immersion challenge of two-week-old catfish fry showed high attenuation levels of *Ei*Δ*evpD*, *Ei*Δ*evpE*, *Ei*Δ*evpG*, *Ei*Δ*evpJ*, and *Ei*Δ*evpK* (19.45%, 15.93%, 13.86%, 15.43%, and 22.13% mortality, respectively) compared to *Ei*WT (100% mortality) (Fig 8C) (*p* < 0.05). On the other hand, attenuation levels of *Ei*Δ*evpF*, *Ei*Δ*evpI*, and *Ei*Δ*evpL* were much lower (94.96%, 82.31%, and 90.41% mortality, respectively), and similar to *Ei*WT (100% mortality). Regardless of their attenuation levels, all mutants showed better protection than sham vaccination (Fig 8D) (*p* < 0.05).

## Discussion

This research involved the development of T6SS mutants, and their performance in various aspects was thoroughly examined. These aspects encompassed their ability to survive and replicate within catfish peritoneal macrophages, their attachment and invasion capabilities in catfish epithelial cells, their resilience against stressors, as well as their virulence and effectiveness in catfish fingerlings and fry. Please refer to Table 3 for a summary of the findings.

**Table 3:** Mutants of T6SS genes in *E*. *ictaluri*.

Mutant	BKA		CCO		Stress Assays				Vaccination			
	Uptake	Survival	Attachment	Invasion	SNP		H_2_O_2_		Virulence		Efficacy	
					BHI	MM19-P	BHI	MM19-P	Fingerling	Fry	Fingerling	Fry
*Ei*Δ*evpA*	**-**	**-**	**-**	**-**	**+**	**-**	**+**	**+**	**+**	N/A	**+**	N/A
*Ei*Δ*evpD*	**-**	**-**	**-**	**-**	**-**	**-**	**-**	**+**	**+**	**+**	**+**	**+**
*Ei*Δ*evpE*	**-**	**+**	**-**	**-**	**-**	**-**	**-**	**+**	**+**	**+**	**+**	**+**
*Ei*Δ*evpF*	**+**	**+**	**+**	**+**	**+**	**-**	**+**	**+**	**+**	**-**	**+**	**+**
*Ei*Δ*evpG*	**+**	**+**	**+**	**+**	**-**	**+**	**+**	**+**	**+**	**+**	**+**	**+**
*Ei*Δ*evpH*	**-**	**-**	**-**	**-**	**+**	**+**	**+**	**+**	**+**	N/A	**+**	N/A
*Ei*Δ*evpI*	**+**	**+**	**-**	**-**	**-**	**+**	**+**	**+**	**+**	**+**	**+**	**+**
*Ei*Δ*evpJ*	**+**	**+**	**-**	**-**	**+**	**+**	**+**	**+**	**+**	**+**	**+**	**+**
*Ei*Δ*evpK*	**-**	**+**	**+**	**-**	**+**	**+**	**+**	**+**	**+**	**+**	**+**	**+**
*Ei*Δ*evpL*	**+**	**+**	**+**	**+**	**-**	**-**	**+**	**+**	**+**	**-**	**+**	**+**
*Ei*Δ*evpM*	**-**	**-**	**+**	**-**	**+**	**+**	**+**	**+**	**+**	N/A	**+**	N/A
*Ei*Δ*evpN*	**-**	**-**	**+**	**+**	**+**	**+**	**+**	**+**	**+**	N/A	**+**	N/A
*Ei*Δ*evpO*	**-**	**+**	**+**	**+**	**+**	**+**	**+**	**+**	**+**	N/A	**+**	N/A

Summary of results: This table provides an overview of the outcomes in various aspects, including survival and replication within catfish peritoneal macrophages, attachment and invasion capabilities in catfish epithelial cells, resistance to stress factors, and virulence and efficacy in catfish fingerlings and fry. Definitions of abbreviations or acronyms used: **BKA:** Bacterial killing assay, involving peritoneal macrophages, uptake at 0 h and survival at 6 h **CCO:** Channel catfish ovary cells, representative of epithelial cells, **Stress Assays:** Cells subjected to stress factors at 8 hours, **SNP:** Sodium nitroprusside, inducing nitrosative stress, **H**_**2**_**O**_**2**_**:** Hydrogen peroxide, causing oxidative stress, **BHI:** Brain-heart infusion broth, a nutrient-rich medium, **MM19-P:** Low-phosphate minimal medium broth at pH 5.5, a minimal medium, **Virulence:** Evaluation through catfish immersion challenge with mutants and *Ei*WT after 21 days, **Efficacy:** Assessment via immersion re-challenge of vaccinated catfish with *Ei*WT after 21 days, **Fingerling:** Refers to six-month-old catfish, **Fry:** Pertains to two-week-old catfish. **(+)** indicates a significant difference compared to *Ei*WT (p < 0.05) and **(-)** denotes no significant difference compared to *Ei*WT (*p* < 0.05). N/A: Abbreviation for "Not Applicable".

The T6SS in *E*. *ictaluri* is composed of an operon containing the genes *evpP* to *evpO* and an additional *hcp2* gene, a homolog to *evpP*, outside the operon (Fig 1). The entire T6SS operon in *E*. *ictaluri* spans a total of 20,784 nucleotides. Although multiple copies of the T6SS is not observed in *E*. *ictaluri*, presence of functionally non-redundant multiple copies of the T6SS in bacterial genomes and existance of plasmid encoded T6SS have been reported [40].

Survival within-host immune cells is crucial for *E*. *ictaluri* as a facultative intracellular pathogen. Studies on other bacteria, such as *Bordetella bronchiseptica* and *Salmonella enterica*, have demonstrated that deleting T6SS genes, such as *tssH* (*clpV*) and *tssM*, enhances intracellular replication [41, 42]. However, in the case of *E*. *ictaluri*, it has been observed that mutations in *tssE*, *tssM*, and *tssH* lead to a decrease in intracellular proliferation inside macrophages [43–46]. The uptake of *Ei*Δ*evpF (tssF)*, *Ei*Δ*evpG (tssG)*, *Ei*Δ*evpI (tssI)*, *Ei*Δ*evpJ (tssJ)*, and *Ei*Δ*evpL (tssL)* inside peritoneal macrophages was higher than the other T6SS mutants in *E*. *ictaluri* (Fig 2). Among these mutants, *Ei*Δ*evpF*, *Ei*Δ*evpG*, and *Ei*Δ*evpJ* increased uptake in peritoneal macrophages. On the other hand, all T6SS mutants and *Ei*WT could not replicate in peritoneal macrophages. This could potentially be attributed to the activated state of catfish peritoneal macrophages, which may limit the intracellular replication of *E*. *ictaluri*. It is worth noting that while deletion of *tssM* and *tssH* resulted in different effects on the fitness and survival of several intracellular bacteria within host immune cells, a mutation in *evpO* (*tssM*) and *evpH* (*tssH*) only resulted in a slight numerical decrease in the intracellular replication of *E*. *ictaluri*. These findings highlight the diverse effects of T6SS mutants on the intracellular lifestyle of *E*. *ictaluri*, underscoring the critical role of the T6SS as an essential secretion system for *E*. *ictaluri*’s adaptation to catfish peritoneal macrophages.

Studies have provided compelling evidence supporting the role of the T6SS in host cell adherence and invasion. Disruption of *tssM* reduced T6SS-mediated adhesion and invasion in various bacteria, including *E*. *coli*, *Campylobacter jejuni*, and *Vibrio parahaemolyticus* [44, 47, 48]. Interestingly, mutation of *tssM* in *Helicobacter hepaticus* resulted in increased adhesion [49]. *Ei*Δ*evpO* homolog of *tssM* has significantly reduced ability in attachment and invasion for CCO cell lines (Fig 3). Similarly, *Ei*Δ*evpN (tssL)* showed significantly less attachment and invasion of the host epithelial cells. Loss of *vgrG* can also affect the attachment and invasion of bacteria for host epithelial cells [50]. *Ei*Δ*evpI*, *a* homolog of *vgrG*, had no significant difference with *Ei*WT regarding attachment and invasion for CCO. Remarkably, *Ei*Δ*evpF (tssF)*, *Ei*Δ*evpG (tssG)*, and *Ei*Δ*evpL (tssJ)* were significantly more adhesive and invasive than *Ei*WT. Although *Ei*Δ*evpK (tssA)* had more attachment ability, its invasion rate was limited. These findings highlight the diverse effects of T6SS mutations on the attachment and invasion abilities of *E*. *ictaluri* in host epithelial cells and underscore the complex interplay between the T6SS and host-pathogen interactions in *E*. *ictaluri*.

The role of the T6SS in stressful conditions highlights its potential importance in the acquisition of crucial metals necessary for bacterial survival within the host [51]. These metals, such as manganese, play a vital role in countering oxidative stress conditions observed in *Burkholderia thailandensis* [52]. Once internalized by host macrophages, bacteria face various stress and killing mechanisms activated by the host to restrict intracellular replication [53]. However, *E*. *ictaluri* has evolved mechanisms to resist these stress and killing mechanisms by upregulating the expression of specific genes [54]. In our study, most T6SS mutants subjected to treatment with SNP and H_2_O_2_ in BHI and MM19-P media (Figs 4–7) showed limited growth under H_2_O_2_-induced stress conditions in both BHI and MM19-P (Figs 6 and 7). Notably, the growth of *Ei*Δ*evpA*, *Ei*Δ*evpH*, *Ei*Δ*evpM*, *Ei*Δ*evpN*, and *Ei*Δ*evpO* mutants was defective in both BHI and MM19-P media. This outcome strongly suggests that T6SS plays a critical role in enabling the survival of *E*. *ictaluri* under stressful conditions. Deletion of specific T6SS mutants may significantly impact the ability of *E*. *ictaluri* to withstand stress conditions that simulate the phagosomal environment of macrophages. These findings underscore the importance of the T6SS in conferring stress resistance and enhancing the survivability of *E*. *ictaluri* in challenging host environments. The ability to counteract oxidative stress and other stressors encountered within the host is crucial for the pathogen’s persistence and successful establishment of infection.

The loss of a functional T6SS can result in a significant reduction in the virulence of pathogenic bacteria. Deletion of *tssM* in *Aeromonas hydrophila*, *C*. *jejuni*, and *Acinetobacter baumannii* has been shown to cause attenuation in mouse models, indicating the crucial role of T6SS in their pathogenicity [47, 55, 56]. Similarly, in *E*. *piscicida evpH*, *evpI*, and *evpC* were found to be essential for establishing an infection in blue gourami [18]. In our study, we investigated the virulence of T6SS mutants in catfish fingerlings and fry. Our data revealed a clear trend: mutants that exhibited complete attenuation in fingerling fish also displayed high levels of attenuation in catfish fry, while mutants with less attenuation in fingerling fish were found to be highly virulent in catfish fry (Fig 8). This consistent observation suggests the reliability and reproducibility of our experiments. Additionally, it highlights the fact that catfish fry possess a less developed immune system compared to fingerling fish [57], rendering them more susceptible to bacterial infections. These findings further support the critical role of the T6SS in the virulence of *E*. *ictaluri*. Deletion of T6SS genes in our study resulted in a significant reduction in virulence in both catfish fingerlings and fry, emphasizing the importance of this secretion system in the pathogenesis of *E*. *ictaluri*.

## Conclusions

In conclusion, this study has elucidated the multifaceted role of T6SS in *E*. *ictaluri*. Firstly, it was revealed that T6SS mutants exhibited varying behaviors in terms of survival and replication within host immune cells, with mutations in *tssE*, *tssM*, and *tssH* leading to a decrease in intracellular proliferation, underscoring the critical role of the T6SS in adaptation to catfish peritoneal macrophages. Secondly, the study demonstrated the intricate relationship between the T6SS and host cell adherence and invasion, with different mutants displaying contrasting abilities in attachment and invasion of host epithelial cells, indicating the complex interplay in host-pathogen interactions. Thirdly, the T6SS was shown to be pivotal in enhancing bacterial survival under stressful conditions, particularly oxidative stress, highlighting its importance in *E*. *ictaluri*’s ability to withstand host defenses. Lastly, the study unequivocally established the indispensable role of the T6SS in the virulence of *E*. *ictaluri*, with mutants displaying reduced virulence in catfish fingerlings and fry, shedding light on its critical contribution to pathogenesis. These collective findings underscore the significance of the T6SS as an essential secretion system for *E*. *ictaluri*’s adaptation, survival, and pathogenicity within the catfish host. The implications of these results extend beyond understanding the basic biology of *E*. *ictaluri* to potentially providing insights for developing effective strategies to control and prevent infections in catfish populations, thus contributing to the broader field of aquaculture and fish health. The diversity of effects observed in this study underscores the complexity of T6SS and its crucial, multifaceted role in the intricate host-pathogen interactions of this bacterium.
